# Adapting depression collaborative care models to increase uptake of computerized cognitive behavioral therapy at the VA: A pilot randomized controlled trial

**DOI:** 10.1016/j.genhosppsych.2025.07.014

**Published:** 2025-07-29

**Authors:** Lucinda B. Leung, Catherine E. Brayton, Sona Hovsepian, Michael A. Karakashian, Karen Chu, Nicholas J. Jackson, Paul G. Shekelle, Alison B. Hamilton, Elizabeth M. Yano, Bruce L. Rollman, Alexander S. Young

**Affiliations:** a Center for the Study of Healthcare Innovation, Implementation & Policy, Veterans Affairs Greater Los Angeles Healthcare System, 11301 Wilshire Blvd., Los Angeles, CA 90073, USA; b Division of General Internal Medicine-Health Services Research, David Geffen School of Medicine, University of California Los Angeles, 10833 Le Conte Ave., Los Angeles, CA 90095, USA; c Department of Psychiatry and Biobehavioral Sciences, UCLA Semel Institute for Neuroscience and Human Behavior, 760 Westwood Plaza, Los Angeles, CA 90095, USA; d Department of Health Policy & Management, UCLA Fielding School of Public Health, 650 Charles E Young Dr., Los Angeles, CA 90095, USA; e Division of General Internal Medicine, University of Pittsburgh School of Medicine, 1218 Scaife Hall, 3550 Terrace Street, Pittsburgh, PA 15213, USA; f Center for Behavioral Health, Media and Technology, University of Pittsburgh School of Medicine, 230 McKee Place, Suite 600, Pittsburgh, PA 15213, USA; g VA VISN 22 Mental Illness Research, Education, and Clinical Center, 11301 Wilshire Blvd., Los Angeles, CA 90073, USA

**Keywords:** Implementation, Primary care, Collaborative care, Depression, Computerized cognitive behavioral therapy

## Abstract

**Objective:**

To examine the feasibility, acceptability, and potential health effects of computerized cognitive behavioral therapy-enhanced collaborative care (cCBT-CC) versus usual primary care (UC).

**Background:**

Internet-based cCBT can effectively treat depression but is not widely used, including in the Veterans Health Administration where it was freely available for veterans. We adapted pre-existing depression collaborative care models using implementation and user-centered design strategies to facilitate cCBT implementation.

**Methods:**

This pilot randomized controlled trial (RCT) included 57 VA primary care patients to cCBT-CC or UC. Participants had Patient Health Questionnaire (PHQ-9) scores of 10+. Those with serious mental illness (e.g., bipolar depression, schizophrenia) and active suicidality were excluded. Intervention patients received tailored Vets Prevail cCBT accompanied by collaborative care manager support, overseen by psychiatry and primary care. UC offered collaborative care services and digital mental health tools at baseline. Feasibility (patient reach, provider adoption, intervention implementation), acceptability (CSQ-8), and potential effectiveness (PHQ-9) data was collected at baseline and 3-months by a blinded study team member.

**Results:**

Participants (cCBT-CC *n* = 29, UC *n* = 28) were 50 years old (mean); 70 % men; 32 % White, 32 % Hispanic, 25 % Black; 21 % homeless-experienced. Mean baseline PHQ-9 scores were 15.1 (SD = 5.0); 39 % reported suicidal thoughts/behaviors. 72 % of 94 primary care providers, from 6 out of the 8 participating clinics, helped support their patients’ participation. cCBT-CC participants received 4 care manager check-ins over 33 days totaling 113 min (64 % clinical; 36 % technical), on average. They completed mean 6.7 out of 11 cCBT lessons. Participants in the cCBT-CC arm experienced a statistically (not clinically) significant decline in the primary outcome of depression (Δ = −2.5; *p* = 0.02) symptoms from pretreatment to posttreatment. There was a greater, albeit non-significant, decrease in PHQ-9 scores among cCBT-CC participants over 3-months, compared to UC participants (Δ = −2.8; 95 % CI = −5.6, −0.01; *p* = 0.05).

**Conclusions:**

cCBT-enhanced collaborative care appeared feasible, acceptable, and possibly effective in treating primary care patients with depression.

## 1. Introduction

Internet-based computerized cognitive behavioral therapy (cCBT) can effectively treat depression but is not widely used in the United States (U.S.). Depression is prevalent and the leading cause of disability globally [1]. Depression is often first identified in primary care, but too few patients initiate and complete evidence-based psychotherapy and medication treatment there [2]. cCBT is a promising alternative treatment modality that has been found to be effective for treating depression in over 40 clinical trials [3–5]. With similar therapy structure and content as face-to-face CBT, cCBT is accessible 24/7 via the internet on desktop computers and mobile devices. cCBT users have shown improvements in depression symptoms and report better quality of life and improved disability levels [6]. cCBT has been incorporated into routine care outside of the U.S., such as the United Kingdom’s National Health Service [7]. cCBT effectiveness still remains unclear in primary care settings, where patient engagement lags, however [5]. To effectively use cCBT to treat U.S. patients with depression, more research is needed to understand how best to implement cCBT in routine primary care settings, including the optimal amount of human support required to ensure patient engagement with these online programs.

Beginning 2008, the VA pioneered Primary Care Mental Health Integration (PCMHI) [8]. Veterans with depression are managed using evidence-based collaborative care models, whereby care managers, mental health clinicians, and primary care providers offer psychotherapy and medication directly in primary care clinics nationally [9]. Then, in 2013, the VA made cCBT available for free to all veterans across the U.S. cCBT is recognized as an alternative treatment option in VA and Department of Defense clinical practice guidelines for major depressive disorder [10]. Specifically, the VA offered Vets Prevail cCBT, a 4-module, 11-lesson online course shown to improve depressive and posttraumatic stress disorder (PTSD) symptoms [11]. Nonetheless, Vets Prevail and other cCBTs struggled with large-scale user reach and user program completion.

In an effort to improve patient reach, increase clinic/provider adoption, and broadly facilitate cCBT implementation, our research team adapted pre-existing VA depression collaborative care models using implementation (e.g., obtaining and using patient/clinician feedback) and user-centered design strategies (e.g., clinician co-design, veteran usability testing). Though available, cCBT programs used by the VA, such as Vets Prevail, had not been systematically supported by clinical services to achieve greater treatment effectiveness and implementation. This support is necessary because individuals with depression are limited by low adherence to cCBT, compared to individuals with other psychiatric conditions [7]. Yet, when delivered with modest clinician support (approximately two hours total), cCBT has fewer drop-outs [12], achieves greater effectiveness [4,7], and has been found to be non-inferior to face-to-face CBT [7,13]. We posit that adapting PCMHI’s collaborative care model to incorporate cCBT with care manager support will enable the opportunity to engage untreated veterans with depression using an alternative treatment modality that is convenient and non-stigmatizing. This pilot randomized controlled trial (RCT) examined the feasibility, acceptability, and potential health effects of cCBT-enhanced collaborative care (cCBT-CC) versus usual care (UC) in VA primary care, in preparation for future effectiveness and implementation trials.

## 2. Methods

This pilot RCT compared cCBT-CC and UC for primary care patients with depression in 8 primary care clinics within the VA Greater Los Angeles (GLA) Healthcare System, one of the nation’s largest VA healthcare systems. UC at these clinics included integrated mental health services (e.g., collaborative care models) and an array of digital mental health tools available at baseline. This study was approved by the VA GLA Institutional Review Board (#1616118) and registered at the U.S. National Library of Medicine’s ClinicalTrials.gov (ID: NCT05050227).

### 2.1. Implementation and user-centered design strategies

This study elicited and used input from key partners in developing cCBT-CC. An advisory board of national VA operational leaders (*n* = 3) and internal and external research experts (*n* = 4) helped guide direct feedback systematically obtained from both veterans and VA clinicians.

#### 2.1.1. Formative interviews and focus groups

Semi-structured interviews were conducted with 16 PCMHI clinicians and staff (nurse care managers, licensed clinical social workers, psychologists, and psychiatrists) in 2018 [14] and with 12 primary care physicians and nurse practitioners (PCPs) in 2020 [15]. Both groups reported general unawareness of but support for cCBT implementation to treat depression as part of existing PCMHI programs. PCMHI clinicians felt cCBT could improve care access and barriers to care (e.g., long wait times for therapy, VA staff shortages). Primary care clinicians discussed possible technical challenges for veterans but reported an overall benefit to having an additional treatment option as guided by established PCMHI care managers.

Additionally, in 2021, four semi-structured, virtual focus groups were conducted with veterans: two with men (*n* = 7) and two with women (*n* = 8) [16]. Few veteran patients were aware of cCBT or its availability through the VA. After learning about it, veterans viewed cCBT as facilitating access to mental health care by addressing treatment barriers including long wait times and perceived limited availability of therapy. Veterans expressed concern about technical aspects of cCBT (e.g., who to contact in an emergency) but thought integrating it into existing primary care models with regular check-ins would alleviate those concerns.

#### 2.1.2. Usability testing with veteran

Individual interviews and usability testing were conducted with a single veteran who completed the Vets Prevail program and was able to meet on a weekly basis to provide feedback, identify problems and frustrations, and report his experiences associated with accessing and utilizing the program. Every week the veteran completed two lessons in the Vets Prevail program and discussed facilitators and challenges he encountered specific to the lessons with our team. Through continued discussions, feedback on usability, accessibility, and content was provided to the software developers and informed the tailoring of our study-specific program instance of Vets Prevail. During this testing period, the veteran was also providing specific information on his satisfaction with all other components of Vets Prevail. Moreover, this allowed our team to understand the time requirement for completing the lessons and ways to improve user performance and satisfaction.

### 2.2. Participants

Eligible participants included veterans who 1) had access to the internet, a telephone, and email communication; 2) were able to read English text on a computer screen; and 3) scored 10 or higher on the Patient Health Questionnaire 9 (PHQ-9). Veterans were excluded if they 1) had active suicidality necessitating emergent treatment or were presently flagged by the VA as being of “high suicide risk”; or 2) carried a diagnosis of serious mental illness (e.g., bipolar disorder, psychosis) or a medical disorder that would prevent/interfere with participation (e.g., dementia/cognitive impairment, terminal illness). Unlike many previous cCBT trials that excluded patients experiencing suicidality [17], this pilot included patients with passive suicidal ideation and who were of low–/moderate-suicide risk, which is prevalent among VA primary care patients. Inclusion criteria were based on a combination of chart review and participant self-report.

### 2.3. Recruitment

We applied a targeted recruitment strategy. Prospective participants were recruited both passively, via flyers placed in primary care and mental health clinics and research buildings, and actively via invitation letters. Recruitment letters were sent to prospective participants identified through local case finding: (1) chart reviews confirmed by primary care providers, which focused on patients who fell out of depression care (i.e., lost to follow up, declined psychotherapy or medication treatment) using previously developed electronic algorithms [18] and; (2) recent and pending consults for depression treatment. Prospective participants either called a recruitment line or were called by a team member to complete a telephone assessment to determine eligibility, to provide verbal consent, and to be scheduled for a baseline assessment.

### 2.4. Sample size and randomization

The primary purpose of this pilot was to assess feasibility and acceptability of trial methods, including recruitment, intervention, and follow-up. Therefore, we aimed to consent a small sample of 64 veterans and to oversample for women, who currently make up 1 in 10 VA users. A research assistant (RA) used the REDCap Randomization Module to assign enrolled participants into two parallel arms, cCBT-CC (intervention) and UC (control), in a 1:1 ratio. Randomization was stratified by PHQ-9 score (i.e., 10–19, >20) to balance arms on depression severity. The RA had no advance knowledge of assignment. Participants were told which arm they were assigned to and those in cCBT-CC were scheduled for their first care manager session.

### 2.5. Intervention: cCBT-enhanced collaborative care (cCBT-CC), including clinician co-design

While agnostic to cCBT program, cCBT-CC employed a tailored study-specific instance of Vets Prevail, the VA-sponsored cCBT program at the time. Vets Prevail delivered 11 sessions of CBT online with accompanying CBT tools (e.g., situation log, SMART goals, activity monitor and scheduler, automatic thoughts record, feelings log). It featured peer support specialist chat support, financial incentives (a $25 online gift card for completing all required components of the program), and safety monitoring that included warm handoffs to the Veterans Crisis Line (a 24/7 crisis hotline for military veterans, service members, their families, and caregivers) [11]. Though the VA made Vets Prevail available to veterans at the time of our study, no participant reported using or being referred to the program prior to enrolling.

Depression care managers are at the core of all collaborative care models, which involve a patient working together with a depression care manager in conjunction with their PCP and consulting psychiatrist. cCBT-CC depression care managers were licensed clinical social workers with master’s degrees and knowledge/experience delivering CBT and were funded by the study. Though depression care managers are part of routine care in VA, none had been specifically trained in supporting cCBT.

cCBT-CC care managers facilitated access to cCBT, promoted and monitored cCBT use, reinforced CBT concepts (during outside CBT session “homework”), and monitored mental health symptoms for each participant. They contacted participants roughly bi-weekly (up to 6 times) for a 15–30-min check-in session via video or phone. Depression care managers reviewed CBT concepts, followed up on assigned homework, as well as employed positive reinforcement and motivation based on CBT principles. The goal was to encourage participants to complete all online cCBT lessons over 3 months and to apply cCBT content and lessons to daily challenges (Appendix 1). While receiving cCBT-CC, intervention arm participant also continued to receive usual care.

Consistent with the collaborative care approach, cCBT-CC depression care managers met with an interdisciplinary team, including a psychiatrist and a primary care physician, on a weekly basis to review the case load, and discuss barriers, progress, and potential challenges with cCBT utilization. A treatment manual was written and revised over the course of the RCT to aid depression care managers in delivering cCBT-CC (Appendix 1). Weekly meetings included clinical updates and participant progress in Vets Prevail. cCBT-CC depression care managers encouraged treatment engagement, focusing on VA primary care and mental health visits. They also met weekly with Vets Prevail software developers to provide continuous direct feedback on the Vets Prevail program, as well as indirect feedback from trial participants. As a result, further changes to the flow of the program (e.g., number of lessons available to complete before a forced stop) were made.

### 2.6. Control: usual care (UC)

UC consisted of depression stepped care, beginning first in VA primary care settings. This typically included medication prescription and follow-up though a patient’s primary care or PCMHI clinicians. UC participants had access to existing VA mental health services, including collaborative care management, and digital mental health tools (e.g., Vets Prevail). However, UC participants may or may not have been uniformly directed to or referred to any digital tools at baseline. Specifically, UC participants did not have access to a depression care manager trained to support their use of cCBT. At the end of the study period all UC participants were given instructions on how to access publicly available Vets Prevail.

### 2.7. Measurements

The primary outcomes for this study were feasibility, acceptability, and health effects on depression. Recruitment feasibility was measured with patient reach (i.e., the number, proportion, and representativeness of participants and reasons for declined or terminated participation) and provider adoption (i.e., PCP support). Provider support of cCBT-CC was conceptualized as assent for the research team to contact appropriate patients and encourage them to try cCBT as a depression treatment, and measured by count of providers who assented. Intervention feasibility was measured with intervention implementation (i.e., number, duration, and type of care manager contact) as well as cCBT usage activity downloaded from Vets Prevail (e.g., registration, enrollment, and lesson completion/duration). These data were supplemented and verified by care manager notes. Acceptability was measured using the Client Satisfaction Questionnaire (CSQ-8) [19], which includes 8 questions rated on a 4-point Likert scale (4 = most satisfied). Additionally, open-ended questions (“What did you like?”, “What did you disklike?”, and “Do you have any suggestions for improvement?”) were asked and answers were transcribed by an RA [20]. Potential health effects on depression was measured using the PHQ-9 [21,22].

Additional health outcomes measured included General Anxiety Disorder (GAD-7) [23]; Posttraumatic Stress Disorder (PTSD) Checklist for DSM-5 (PCL-5) [24]; and PROMIS-10 Global Health, which has physical and mental health components [25]. Patient and behavioral activation, as measured via Patient Activation Measure (PAM) [26] and Behavioral Activation for Depression Scale, Short Form (BADS-SF) [27], were hypothesized mediators. Finally, we asked participants the following questions about their health services use: “During the past 6 months, how many times did you…” (1) “…go to any mental health provider, including psychiatrists, psychologists, social workers, psychiatric nurses, or counselors?”, (2)”… go to a general medical provider like a family doctor, general internist, gynecologist, nurse or physician assistant?”, (3) “take any prescribed medications for mental or emotional problems?” A self-reported measure of service use was used since veterans may receive care outside the VA (e.g., community care, health services from other insurance).

All trial participants were asked to complete self-reported assessments at 2 timepoints: baseline (30 min) and 3-months (45 min) post enrollment. Though study assessments were validated for self-administration, one trained RA administered each assessment by phone. Allocation was concealed from the RA, who was only unblinded at the end of the 3-month assessment when additional questions were asked of the cCBT-CC participants about program acceptability. All participants completed the same baseline assessment, which included measures of overall health, mental health, use of health services, and demographics. Three-month follow-up assessments for UC were nearly identical to the baseline survey (excluding demographics); cCBT-CC follow-up assessments included the measures of program acceptability. Participants received a $30 gift card after completing the baseline assessment and a $45 gift card after completing the 3-month follow-up assessment as compensation for their time.

### 2.8. Data analyses

To assess feasibility and acceptability, we used descriptive and summary statistics. For effectiveness analyses, we applied intention to treat (ITT) principles to assess between-group differences in change over time for (1) depression severity, (2) anxiety severity, (3) PTSD severity, (4) health-related quality of life, (5) patient and behavioral activation, and (6) mental health and primary care visits. Mixed effects models (linear, logistic) and negative binomial regression were specified with group (cCBT-CC vs UC) and time (3-month versus baseline) fixed effects and their interaction. A random intercept for patient was specified to account for correlation among repeated measurements within-patients. We also assessed psychotropic medication use, which did not vary over time, between groups using logistic regression. We additionally present standardized effect sizes (Cohen’s d) to provide a sense of the magnitude between-group differences observed. This pilot study intended to describe feasibility and acceptability outcomes and was not sufficiently powered to examine effectiveness outcomes. Lastly, we compared cCBT-CC and UC participants on the percentage who experienced a clinically significant remission in depression, that is PHQ-9 < 10 (full) or < 5 (partial), using a X^2^ test. Statistical significance was evaluated using a two-sided alpha level of 0.05. All analyses were conducted in Stata 18.0 (College Station, TX, USA).

Open-ended question responses were analyzed using matrix analysis techniques. A templated summary was generated for each respondent and summaries were transferred into a matrix, created in Microsoft Word (Redmond, WA, USA). Two team members independently reviewed the matrix to identify potential themes, then compared findings and came to consensus about the results [28]. Nearly all themes were identical across the two analysts’ results.

## 3. Results

### 3.1. Participant flow

**Fig. 1.**
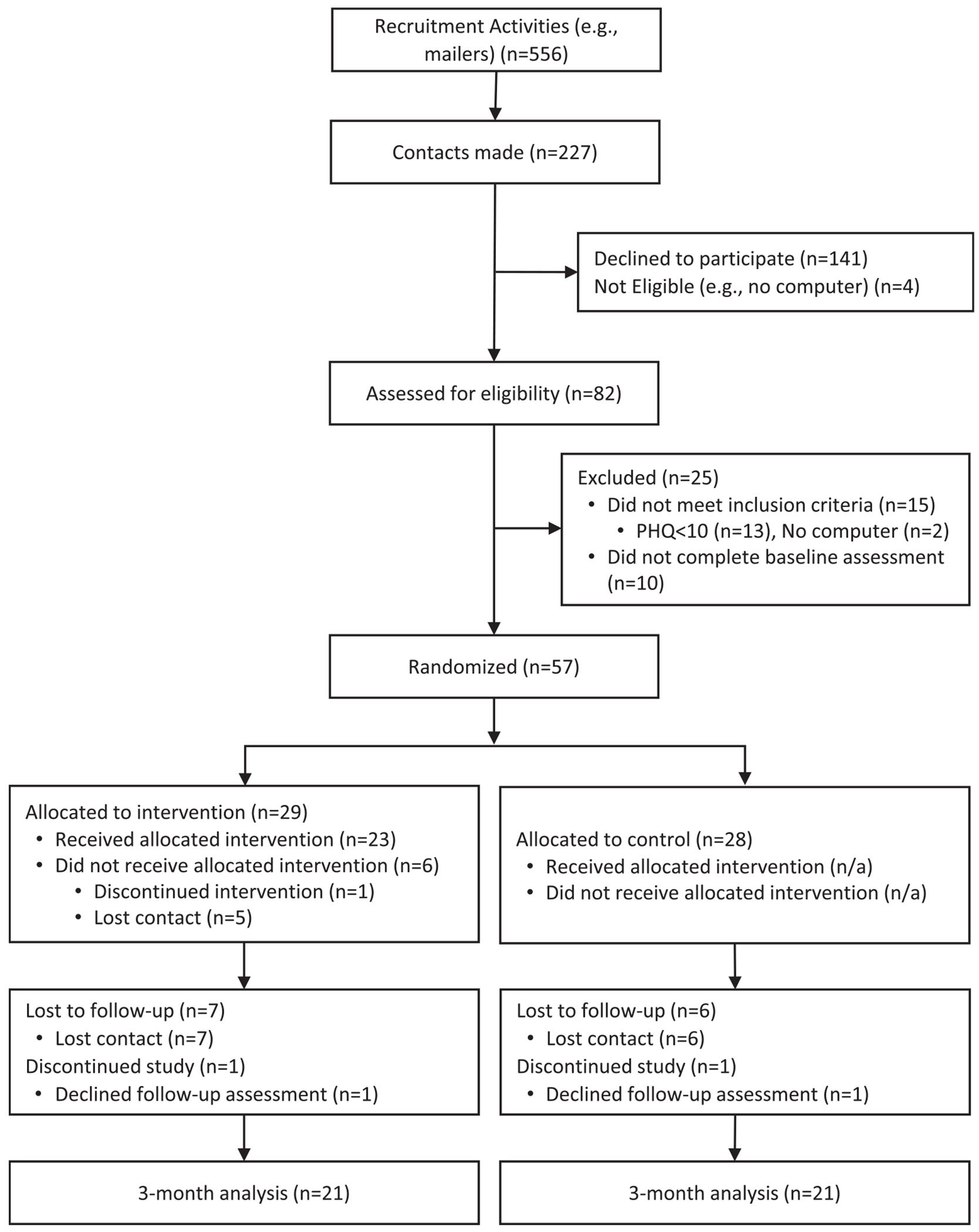
CONSORT participant flow diagram.

Participants were recruited on a rolling basis between November 2021 and February 2023 with follow-up between April 2022 and May 2023. Given that our target population was veterans with depression who were not currently in mental health treatment, we recruited widely to account for low interest among potential participants. We identified 556 potential participants via case finding activities (e.g., mailers and flyers), contacted 227 by phone, assessed 82 for eligibility, enrolled 57 and randomized them into UC (*n* = 28) or cCBT-CC (*n* = 29) arms. Of the 29 intervention arm participants, 23 received the intervention (Fig. 1). Recruitment efforts were intentionally focused on women veterans to achieve 30 % oversampling of this fast-rising VA patient minority group. Utilizing two types of case finding for recruitment (chart reviews and recent and pending consults) led to increased recruitment of women veterans and quicker recruitment overall. When target recruitment numbers were reached, the trial ended. Retention in study assessments was high with 74 % completing posttreatment assessments.

Participants had a mean age of 50 years, were 70 % men, 32 % White, 32 % Hispanic, 25 % Black, and 21 % homeless-experienced (Table 1). Mean baseline PHQ-9 scores were 15.1 (SD = 5.0) and 39 % reported suicidal thoughts or behaviors. About a third of participants had a positive alcohol (35 %, AUDIT-C) or cannabis (33 %) screen, and 18 % of control arm participants reported using drugs.

### 3.2. Feasibility of cCBT implementation

72 % of 94 primary care providers (e.g., physicians, nurse practitioners), from 6 out of the 8 participating clinics, encouraged their patients’ participation in cCBT-CC. Intervention arm participants received 4 care manager check-ins over 33 days totaling 113 min (64 % clinical; 36 % technical) of check-in time, on average, akin to evidence-based collaborative care. They completed mean 6.7 of 11 cCBT lessons and spent a median of 17.7 min per lesson. Of the 29 intervention arm participants, 18 (62 %) completed at least half (≥5) of the lesson and 12 (41 %) completed all the lessons. (Table 2).

### 3.3. Acceptability of cCBT-CC program

Participants rated cCBT lessons favorably, CSQ-8 mean 3.27 out of 4. They found Vets Prevail cCBT to be acceptable and easy to understand. In response to open ended questions, one participant stated, “I liked the videos, they were detailed. They were quick and easy to get into.” Participants also liked meeting with the depression care manager, and one noted, “I liked the interaction because it held me accountable” and “[the depression care manager] was just really amazing and was able to feed into what I needed…”. In contrast, participants were frustrated by technical issues and the focus on combat trauma. One participant thought “contacting your [depression care manager] on there is clunky sometimes. They had to call me most of the time.” Another participant mentioned that the program felt more tailored toward recently discharged veterans: “[The program] slid more toward people who just exited the military.” Lastly, participants had several suggestions including: broadening the program so it was more applicable to “older Vets” and women veterans, improving technology including the ability to look at the program tools (e.g., activity tracker) during a video chat with the depression care manager, and ensuring continuity in care managers – “it’s important to speak with one person beginning to end.”

### 3.4. Measured health outcomes

At baseline, there were no significant differences in the primary or secondary health outcomes between UC (*n* = 28) and cCBT-CC (*n* = 29) arms. Participants in the cCBT-CC arm experienced a statistically significant decline in the primary outcome of depression (Δ = −2.5; 95 % CI = −4.4, −0.05; *p* = 0.02) from pretreatment to posttreatment; however, there was a non-significant decrease in PHQ-9 scores among cCBT-CC participants from baseline to 3-month follow-up, compared to UC participants (Δ = −2.8; 95 % CI = −5.6, −0.01; *p* = 0.05). The magnitude of differences in PHQ-9 scores is also below the threshold of a minimal clinically significant difference. Although participants in the cCBT-CC arm experienced a statistically significant decline in only the secondary outcome of PTSD (Δ = −5.6; 95 % CI = −11.1,−0.02; *p* = 0.04) from pretreatment to posttreatment, we did not find statistically significant improvements in PTSD, anxiety, or quality of life scores, when comparing cCBT-CC to UC arms. There were also no significant effects between arms for our hypothesized mediators of patient activation and of behavioral activation. There were no statistically significant differences in reported number of visits to general medical and to mental health providers, nor in reported use of psychotropic medications (which did not vary from baseline during the study), across participants in both arms (see Table 3 for full results). There were no significant differences in any health outcome between UC (*n* = 21) and cCBT-CC arms (n = 21) either. Four (19 %) participants in the cCBT-CC arm achieved partial or full remission of depression (PHQ-9 < 10 or < 5, respectively) over 3 months, versus 1 (5 %) in the UC arm (X2 = 2.04; *p* = 0.15).

## 4. Discussion

In our pilot RCT, cCBT-enhanced collaborative care appeared feasible and acceptable for use to treat veterans with depression in primary care. Research procedures were confirmed to be feasible as the trial reached diverse participants who were largely representative in age, sex/gender, and race/ethnicity of VA primary care populations in our large, metropolitan region. Findings may contribute to cCBT knowledge on engagement among older adults and among racial/ethnic minorities [29]. Moreover, we are not aware of any trials that specifically examine cCBT use among women veterans, who typically have complicated depression and multiple psychiatric comorbidities [30,31]. Veterans were pragmatically recruited and had high rates of concurrent alcohol misuse, suicidal ideation, and housing instability, typical of VA GLA and the most vulnerable primary care patients. To our knowledge, no cCBT trials have engaged a sizeable number of homeless-experienced veterans as participants. Importantly, this pilot also demonstrated high buy-in from primary care clinicians across heterogeneous clinic sites, including from (non-academic) community-based sites; the majority were agreeable to and participated in this new care model. Veterans received care-manager support similar to existing VA-based collaborative care models, i.e., 2 h over 4 visits, on average. In the cCBT-CC arm, veterans engaged in and completed, on average, more than half of cCBT lessons with 41 % completing all lessons, a marked improvement from self-guided cCBT use as reported by Vets Prevail (e.g., <20 % completion rate in VA Los Angeles in 2018). Future cCBT programs could be adaptive in nature, such as tailored lesson content based on disease comorbidity (e.g., supplementary CBT for insomnia) or shortened program length with symptom improvement.

Although this pilot RCT was not adequately powered to detect statistical significance, we observed promising signals of depression improvement. Meticulous coordination of existing VA mental health resources (i.e., cCBT + collaborative care) demonstrated 3-fold the number participants in the cCBT-CC arm achieving partial or full remission over 3 months, compared to the UC arm. A larger hybrid effectiveness-implementation trial is still warranted to confirm our preliminary findings. Nonetheless, this study has added to the few clinical trials on computerized psychological interventions for veterans [32,33]. Prior cCBT trial results were most often derived from participants who may not generalize to veterans, whom are older and have more chronic health conditions [17]. Furthermore, our study helped to understand cCBT effectiveness at the primary care population level, where results have ranged from ineffective (without support) to moderately effective (with therapist support, of which there is a shortage in primary care) [5]. Unlike prior primary care-based cCBT trials, this pilot RCT included a control arm that offered robust usual care services, including cCBT (albeit, self-guided) and collaborative care services for treating depression [34,35]. Finally, this pragmatic trial included vulnerable veterans (e.g., those who previously declined depression care, those with low−/moderate-suicide and/or co-occurring behavioral health conditions, homeless-experienced veterans), whom are known to have high rates of co-occurring mental illness, which may have dampened our effect sizes; nonetheless, our trial did not experience any adverse events.

While preliminary results on depression outcomes concur with existing literature [36], our study contributes most greatly through our demonstration of implementation and user-centered design strategies to incorporate online cCBT into clinical care at the broad-based population level. This study employed multiple strategies highlighted by the Expert Recommendations for Implementing Change (ERIC) project, specifically facilitation; assessing for readiness and identify barriers and facilitators; obtaining and using patient/clinician feedback [14–16]; involving patients/clinicians in the implementation effort; conducting educational meetings; developing educational materials (Appendix- cCBT-CC Treatment Manual) [37]. Based on the Consolidated Framework for Implementation Research [38] (CFIR), we obtained input from persons who have influence and/or power over the outcome of implementation efforts, including “recipients” who benefit from the innovation (i.e., veterans) and “deliverers” of the innovation (i.e., VA clinicians, organizational leaders). Furthermore, by actively involving veterans and VA clinicians throughout the design lifecycle of cCBT technology, we leveraged user centered design strategies to ensure cCBT is more able to incorporate the needs, values, and lived experiences of potential users [39]. This pilot RCT was designed to mirror a hybrid type 1 effectiveness-implementation trial, aiming to gather some effectiveness data on cCBT and to understand its potential for implementation in real-world settings [40]. Future trials of cCBT-CC can build on and test for the optimal selection of implementation strategies, so that cCBT can be incorporated into existing PCMHI models and then disseminated across VA clinics nationally.

This pilot trial primarily assessed and found it feasible and acceptable to use cCBT as part of existing collaborative care models that treat depression among primary care populations. We are limited to drawing only preliminary conclusions regarding the health effects of cCBT-CC, because the pilot’s small sample limit our confidence in the effect size estimates [41,42]. Additional limitations of this small pilot included: only one Vets Prevail user gave feedback during cCBT-CC developmental phase, lack of data on collaborative care visits or digital mental health tools used as part of usual care, acceptability (using CSQ-8) was only measured for cCBT-CC participants, follow-up was only for 3 months. However, this study is strengthened by its randomized assignment to condition, use of a treatment manual for cCBT-CC, and testing in a real-world, practical setting. More research is needed to test effectiveness and implementation strategies for cCBT-CC as a way to expand access to cCBT for depression. In conclusion, cCBT-enhanced collaborative care appears to be feasible and acceptable for increasing engagement in depression treatment in primary care settings. There is potential for it being a viable and effective way to overcome treatment barriers such as long wait times and veteran preference for psychotherapy and improve VA’s capacity to deliver evidence-based psychotherapy.

## Figures and Tables

**Table 1:** Baseline patient demographics and behavioral health.

	Total (n)%	Control (n)%	Treatment (n)%
Patient Demographics			
Age, years (mean ± SD)	(57) 49.9 ± 16.3	(28) 49.8 ± 15.1	(29) 49.9 ± 17.6
Gender (% Female)	(17) 30 %	(9) 32 %	(8) 28 %
Marital Status (% Currently Married)	(12) 21 %	(5) 18 %	(7) 24 %
Race-Ethnicity			
Hispanic	(18) 32 %	(8) 29 %	(10) 34 %
White	(18) 32 %	(9) 32 %	(9) 31 %
Black or African American	(15) 26 %	(8) 29 %	(7) 24 %
Asian	(3) 5 %	(2) 7 %	(1) 3 %
Native Hawaiian or Other Pacific Islander	(1) 2 %	(0) 0 %	(1) 3 %
Other	(2) 4 %	(1) 4 %	(1) 3 %
Education			
Some high school, but did not graduate	(1) 2 %	(1) 4 %	(0) 0 %
High School Diploma or GED	(32) 56 %	(15) 54 %	(17) 59 %
Some college or 2-year degree	(16) 28 %	(8) 29 %	(8) 28 %
4-year college degree	(8) 14 %	(4) 14 %	(4) 14 %
Work Status			
Working full-time	(24) 42 %	(9) 32 %	(15) 52 %
Working part-time	(7) 12 %	(3) 11 %	(4) 14 %
Not working	(26) 46 %	(16) 57 %	(10) 34 %
Living Situation			
Own a home or condo	(14) 25 %	(7) 25 %	(7) 24 %
Pay rent/Live in subsidized housing	(30) 53 %	(13) 46 %	(17) 59 %
Homeless-experienced	(12) 21 %	(7) 25 %	(5) 17 %
Prefer not to answer	(1) 2 %	(1) 4 %	(0) 0 %
Behavioral Health & Function			
Columbia Suicide Severity Rating Scale			
No risk	(35) 61 %	(16) 57 %	(19) 66 %
Low risk	(12) 21 %	(6) 21 %	(6) 21 %
Medium risk	(9) 16 %	(6) 21 %	(3) 10 %
High risk	(1) 2 %	(0) 0 %	(1) 3 %
Alcohol Use Disorders Identification Test-Consumption (% positive alcohol screen)	(20) 35 %	(9) 32 %	(11) 38 %
Cannabis (% use in past 3 months)	(19) 33 %	(10) 36 %	(9) 31 %
Drug and medicine misuse (% yes)*	(5) 9 %	(5) 18 %	(0) 0 %
Use of psychotropic medications	(27) 47 %	(16) 57 %	(11) 38 %

* p = 0.02; Do you use medicines on you own, that is without a doctor’s prescription, in greater amounts or longer than prescribed (e.g., painkillers, stimulants, sedatives, marijuana, cocaine)?

**Table 2:** Intervention Feasibility.

	n = 29
cCBT Care Management	
Days with care manager (mean, SD)	33 (26.3)
Number of care manager check-ins (mean, SD)	4.0 (2.8)
Clinical minutes, total (mean, SD)	71.5 (56.0)
Clinical minutes, per check-in (mean, SD)	15.7 (6.9)
Technical minutes, total (mean, SD)	41.3 (37.8)
Technical minutes, per check-in (mean, SD)	12.4 (8.9)
cCBT Program Use	
Lessons completed (mean, SD)	6.7 (4.9)
Number participants who completed <5 lessons	11 (37.9 %)
Number participants who completed ≥5 lessons	18 (62.1 %)
Number participants who completed all lessons	12 (41.4 %)
Minutes using cCBT (median)	141.7
Minutes using cCBT, per lesson (median)	17.7

**Table 3:** Effects of cCBT-CC and UC on depression and secondary health outcomes.

All Participants	Usual Care (UC)					cCBT-Enhanced Collaborative Care (cCBT-CC)					Difference-in-difference		
	Baseline n = 28 (mean, SD)	3-months n = 21 (mean, SD)	Change	95 % CI	p	Baseline n = 29 (mean, SD)	3-months n = 21 (mean, SD)	Change	95 % CI	p	Estimate	95 % CI	p
Behavioral Health & Functioning													
Patient Health Questionnaire, PHQ-9	14.6 (0.9)	15.0 (1.1)	0.4	−1.6 to 2.4	0.73	15.4 (0.9)	13.0 (1.0)	−2.5	−4.4 to −0.5	0.02*	−2.8	−5.6 to −0.0	0.05
Patient Activation Measure, PAM	37.9 (0.9)	38.8 (1.0)	1.0	−0.7 to 2.7	0.25	37.1 (0.8)	36.7 (1.0)	−0.3	−2.0 to 1.4	0.70	−1.3	−3.7 to 1.6	0.28
PTSD Checklist for DSM-5	38.7 (3.0)	33.3 (3.7)	−5.4	−10.9 to 0.1	0.05	40.3 (2.9)	34.7 (3.7)	−5.6	−11.1 to −0.2	0.04*	−0.2	−8.0 to 7.5	0.95
General Anxiety Disorder, GAD-7	12.5 (0.9)	10.7 (1.1)	−1.7	−3.6 to 0.1	0.07	12.3 (0.9)	10.6 (1.1)	−1.7	−3.6 to 0.1	0.07	0.0	−2.6 to 2.6	1.00
Behavioral Activation for Depression Scale-SF	20.4 (1.8)	22.0 (2.0)	1.6	−2.4 to 5.5	0.44	20.2 (1.7)	20.0 (2.0)	−0.2	−4.1 to 3.7	0.93	−1.7	−7.3 to 3.8	0.54
PROMIS-10 Global Health													
Physical Health	39.1 (1.6)	38.4 (1.8)	−0.7	−3.9 to 2.54	0.69	37.5 (1.6)	37.3 (1.8)	−0.2	−3.4 to 2.9	0.88	0.4	−4.1 to 4.9	0.86
Mental Health	35.5 (1.4)	37.4 (1.6)	1.9	−1.3 to 5.1	0.24	33.7 (1.3)	36.5 (1.5)	2.8	−0.4 to 6.0	0.09	0.9	−3.6 to 5.4	0.70
Health Services Use													
No. of mental health visits	5.8 (1.6)	9.0 (2.4)	3.1	−0.9 to 7.2	0.13	5.2 (1.4)	4.9 (1.3)	−0.3	−3.0 to 2.4	0.17	−3.4	−8.3 to 1.4	0.17
No. of general health visits	1.3 (0.1)	1.9 (0.3)	0.1	0.1 to 1.1	0.0*	1.4 (0.2)	1.4 (0.3)	0.0	−0.6 to 0.6	0.15	−0.6	−1.3 to 0.2	0.15

Mixed effects models (linear, logistic) and negative binomial regression were specified with group (cCBT-CC vs UC) and time (3-months versus baseline) fixed effects and their interaction. A random intercept for patient was specified to account for correlation among repeated measurements within-patients.

## Data Availability

Data will be made available on request.

## Appendix 1. cCBT-CC treatment manual

### Preface

#### Steps Prior to Offering Participation in cCBT

- Ensure Veteran has:
  - Email address
  - Access to internet
  - Access to a computer, tablet, or smartphone

#### Steps Prior to Clinician Check In Session

- Call Veteran one day before scheduled appointment. For Monday appointments, call Veterans on Friday.
- Ensure Veteran has created an account and has his/her login and password ready prior to the session.
- Preparation for the session:
  - Refer to **program outline** to reference what session (s) should be delivered/reviewed**.**
  - Be sure to know the key concepts for the session (s) to be reviewed.
  - Review protocol for management of suicide risk.
- Know how to contact the help desk and share contact information with the Veteran as well.
- Have a backup plan to contact the Veteran if VetsPrevail isn’t working correctly (e.g., Zoom, Teams, patient telephone number).

#### Session Tips

- Verify Veteran’s location at the beginning of each session and confirm call back number.
- You may need to redirect but use motivational interviewing techniques to explore topics.
- It is normal for Veterans bring the difficulties of the military to civilian life- some difficulties are a very normal response to circumstances of military life.
- Identifying and preparing to face challenges is a mark of strength and resilience.
- Note sessions completed (optional) + goals from the sessions.
- Review Veteran’s engagement/utilization of mental health services (at the VA).
  - Provide contact information as needed to mental health services.

1. SESSION GUIDELINES: Welcome and Account Set-up
  - Introductions
    - Give introductions and thank Veteran for joining
    - Review agenda for meeting
      [*about 2*–*3* min]
  - Account set up and program basics
    - Determine what kind of device Veterans is using (this may change how the account set up / functions of the program work).
    - Walk through setting up account. Make sure Veteran has successfully set up account. Remind Veteran to write down username and password and bookmark program webpage (if applicable).
    - Walk through basic functions of the program:
      - How to start a new lesson
      - How to review old lessons
      - How to access tools & activities
      - How to connect with a care manager (if done within program)
      - How to cash in rewards (if applicable)
      - How to contact help desk if technical support is needed
    - Make sure to have help desk contact information readily available
      - Questions from Veteran
        [*about 10*–*15* min]
  - Reviewing the key concepts of the program
    - Introduce the role of the depression care manager:
      - Frequency and length of depression care manager check-in
      - Content of check-in:
        - General check-in and progress tracking
        - Review lessons and answer questions
        - Establish goals specific to lesson content
      - Will work together with a PCP and psychiatrist to ensure your progress
    - Introduce content of computerized cognitive behavioral therapy:
      - It is a program based in Cognitive Behavioral Therapy (CBT)
      - It will help you learn tools to regulate emotions
      - You will complete tools to practice lesson content
      - You can connect with other Veterans who have dealt with similar problems (if applicable)
      - You will earn points for the lessons you complete and can use them toward a gift card (if applicable)
    - Check-in on Veteran’s healthcare utilization at VA.
      [*about 5*–*7* min]
  - Next Steps:
    - Identify which sessions need to be completed prior to next appointment.
      - Complete Introductory Program: Levels 1 and 2
    - Questions from veteran?
    - Schedule next appointment or repeating weekly appointments as Veteran is able. Provide contact information should there be any questions between now and next appointment.
      [*about 3*–*5* min].

1. SESSION GUIDELINES: Levels 1 and 2
  - Check in with the Veteran
    - Review Veteran’s PHQ-9/GAD-7 scores before session.
    - How are you feeling today?
    - Did you watch your videos and finish your activities (tools, logs, if applicable) for each session?
      *[about 3*–*5 min]*
  - Begin by reviewing the key concepts identified in the session (s)
    - Define key concepts:
      Level 1 – Intro (Part 1): Overcoming Challenges
      - It is a tool to help us understand and take control of our thoughts, behaviors, and emotions in order to deal with everyday problems. Help you develop practical skills.
      - CBT was developed by doctors and there is 50 years of clinical research to support it.
        Level 2 – Intro (Part 1): Overcoming Challenges
      - Thoughts: How we perceive our environment, others, and ourselves. Helps us draw conclusions about our life.
      - Emotions: Feelings we experience based on our perception of the situation
      - Behaviors: How we interact as a result of these perceptions and feelings
        Situation Log Tool (*will be built into system and come up as part of lessons plans*).
        *[about 5*–*8* min*]*
  - Discussion Points:
    - What is the takeaway message that you thought was most helpful for you from levels 1 and 2?
    - Which part of the lessons are you able to apply to your day-to-day activities?
    - Explore the Veteran’s goals from this session and identify action items for the week, if applicable
      - Is the Veteran inspired to try something new based on today’s topic? If yes, assign the optional tools/activities.
    - Draft action plan utilizing content from levels 1 and 2
    - Questions from veteran?
      *[about 5*–*8 min]*
  - Next Steps
    - Confirm next appointment; provide contact information should there be any questions between now and next appointment.
    - Identify which sessions need to be completed prior to next appointment.
      *[about 3*–*5 min].*
2. SESSION GUIDELINES: Levels 3, 4, and 5
  - Check in with the Veteran
    - Review Veteran’s PHQ-9/GAD-7 scores before session.
    - How are you feeling today?
    - Did you watch your videos and finish your activities for each session?
      *[about 3*–*5 min]*
  - Begin by reviewing the key concepts identified in the session (s)
    - Define key concepts:
      Level 3 – Intro (Part 2): Overcoming Challenges
      - Thoughts, behaviors, and emotions form a mutually interactive system. Each one affects and is affected by the other. (See level 2 for definitions of above)
        [Guided Introduction to a VA service determined by the user’s profile].
        Level 4 – Intro (Part 2): Overcoming Challenges
      - Check - > (Monitor your thoughts, behaviors, and emotions) 2. Identify what’s dysfunctional - > 3. Deal with the dysfunctions effectively
      - Become aware of and monitor our human processes in order to stop and perform maintenance.
      - *SMART Goal Tool*- [will be built into system and come up as part of lessons plans]
        Level 5 – Getting Down the Basics Q&A- 6 multiple choice questions
      - Dysfunctional Thought Processes- unhelpful, unproductive, unrealistic, unreasonable
      - Learn to monitor and identify dysfunctions
      - Negative Feedback loops- one reinforces the other
        *[about 5*–*8* min*]*
  - Review the Veteran’s Goals for the session
    *SMART Goal Tool*- [will be built into system and come up as part of lessons plans]
  - Discussion Points:
    - What is the takeaway message that you thought was most helpful for you from lessons 1 and 2?
    - Which part of the lessons are you able to apply to your day-to-day activities?
    - Explore the Veteran’s goals from this session and identify action items for the week, if applicable.
      - Is the Veteran inspired to try something new based on today’s topic? If yes, assign the optional tools/activities.
    - Draft action plan utilizing content from lessons 3,4, and 5
    - Questions from veteran?
      *[about 5*–*8 min]*
  - Next Steps
    - Confirm next appointment; provide contact information should there be any questions between now and next appointment.
    - Identify which sessions need to be completed prior to next appointment.
      *[about 3*–*5* min*].*
3. SESSION GUIDELINES: Behaviors Program – Levels 6 and **7**
  - Check in with the Veteran
    - How are you feeling today?
    - Did you check in with your peer coach?
    - Review/Reflect on the PHQ-9 and GAD-7
    - Did you watch your videos and finish your activities for each session?
      *[about 3*–*5 min]*
  - Begin by reviewing the key concepts identified in the session (s)
    - Define key concepts:
      Level 6 – Behaviors (Part 1): A closer Look at Your Actions
      - Relationship between motivation and activity- the two feed into and reinforce one another
      - Commit to getting active and motivation to stay active will follow
        - Increased activity- > improved mood
      - Pleasure: Amount of enjoyment we experience from an activity
      - Mastery: Ability to perform activity, and sense of accomplishment from completing it
        *Activity Monitor Tool*- monitor pleasure and mastery in each activity.
        Level 7 – Behaviors (Part 2): A Closer Look at Your Actions
      - Identifying paths of dysfunctional behavior
      - Identifying imbalances with the pie chart- insert photo here from Vets Prevail (ok to add photos)
      - Plan Healthy Activities
        *Activity Scheduler Tool*- schedule an activity.
        *[about 5*–*8* min*]*
  - Discussion Points:
    - What is the takeaway message that you thought was most helpful for you from lessons 6 and 7?
    - Which part of the lessons are you able to apply to your day-to-day activities?
    - Explore the Veteran’s goals from this session and identify action items for the week, if applicable.
      - Is the Veteran inspired to try something new based on today’s topic? If yes, assign the optional tools/activities.
    - Draft action plan utilizing content from lessons 6 and 7
    - Questions from veteran?
      *[about 5*–*8 min]*
  - Next Steps
    - Confirm next appointment; provide contact information should there be any questions between now and next appointment.
    - Identify which sessions need to be completed prior to next appointment.
      *[about 3*–*5 min].*
4. SESSION GUIDELINES: Thoughts Program – Lessons 8 and 9
  - Check in with the Veteran
    - How are you feeling today?
    - Did you check in with your peer coach?
    - Review/Reflect on the PHQ-9 and GAD-7
    - Did you watch your videos and finish your activities for each session?
      *[about 3*–*5* min*]*
  - Begin by reviewing the key concepts identified in the session (s)
    - Define key concepts:
      Level 8 – Thoughts (Part 1): Think Bigger, Think Better
      - The way we interpret the situation affects how we feel and react to it.
      - Thoughts = lens through which we view the world.
      - Reasonable and Productive versus Irrational and Unproductive thinking
      - Human thought processes are complicated:
        - Identifying automatic thoughts can help you develop more productive approaches to everything you do.
      - An example of an automatic thought is breathing
        - The best way to counter dysfunctional thinking is to identify and become aware of when your mood is becoming worse or you’re behaving in unproductive ways.
          *Automatic thought record tool*- By writing out these situations you will eventually be able to rework the way you think about things and create healthier thought processes.
          Level 9 – Thoughts (Part 2): Think Bigger, Think Better
      - Learn how to examine your thoughts to catch where things commonly go haywire.
      - Recognize Cognitive Errors- bad habits of thinking
      - Identifying dysfunctional thoughts is the first step in challenging/preventing them
      - Our thinking can go unnoticed unless we take the time to reflect
        *[about 5*–*8* min*]*
  - Discussion Points:
    - What is the takeaway message that you thought was most helpful for you from lessons 8 and 9?
    - Which part of the lessons are you able to apply to your day-to-day activities?
    - Explore the Veteran’s goals from this session and identify action items for the week, if applicable
      - Is the Veteran inspired to try something new based on today’s topic? If yes, assign the optional tools/activities.
    - Draft action plan utilizing content from lessons 8 and 9
    - Questions from veteran?
      *[about 5*–*8 min]*
  - Next Steps
    - Confirm next appointment; provide contact information should there be any questions between now and next appointment.
    - Identify which sessions need to be completed prior to next appointment
      *[about 3*–*5 min].*
5. SESSION GUIDELINES: Emotions Program – Lessons 10 and 11
  - Check in with the Veteran
    - How are you feeling today?
    - Did you check in with your peer coach?
    - Review/Reflect on the PHQ-9 and GAD-7
    - Did you watch your videos and finish your activities for each session?
      *[about 3*–*5 min]*
  - Begin by reviewing the key concepts identified in the session (s)
    - Define key concepts:
      Level 10 – Emotions (Part 1): Know and Control How You Feel
      - Explore and understand your emotions and what causes them. Learn strategies to control your feelings so they don’t control you
      - We experience emotions through our minds and bodies
      - Just being conscious of our emotions helps us deal with our feelings in a healthy way
      - Emotional reasoning- draw the line between how we feel and how we reason through our feelings.
      - Distinguishing between how we feel and the thoughts we are having is critical
      - Emotions (Anger, Disgust, Fear, Joy, Sadness). Feelings (more specific and varied, the ways we personally experience emotions). Mood (general prolonged emotional state).
      - Describing and categorizing feelings is a big step. Rate our feelings they are not an all or nothing experience.
        *Feelings Log*- Understanding patterns of intense feelings will help you to identify the situations that make us feel this way.
        Level 11 – Emotions (Part 2): Know and Control How You Feel
      - Practical Techniques
      - Awareness Exercise- it takes practice to recognize feelings
        - Acknowledge and accept feelings as natural
        - Difficult emotions are unavoidable
        - Getting in tune with emotions isn’t fixing them right away
        - Understanding emotions to respond to them in healthy ways
      - Relaxation Techniques- controlled breathing, simple meditation, progressive muscle relaxation technique
        *[about 5*–*8* min*]*
  - Discussion Points:
    - What is the takeaway message that you thought was most helpful for you from lessons 10 and 11?
    - Which part of the lessons are you able to apply to your day-to-day activities?
    - Explore the Veteran’s goals from this session and identify action items for the week, if applicable
      - Is the Veteran inspired to try something new based on today’s topic? If yes, assign the optional tools/activities.
    - Draft action plan utilizing content from lessons 10 and 11
    - Questions from veteran? Congratulations for completing the program!
      *[about 5*–*8 min]*.
